# Improving automatic liver tumor segmentation in late-phase MRI using multi-model training and 3D convolutional neural networks

**DOI:** 10.1038/s41598-022-16388-9

**Published:** 2022-07-18

**Authors:** Annika Hänsch, Grzegorz Chlebus, Hans Meine, Felix Thielke, Farina Kock, Tobias Paulus, Nasreddin Abolmaali, Andrea Schenk

**Affiliations:** 1 Fraunhofer Institute for Digital Medicine MEVIS, Bremen, Germany; 2 Medical Image Computing Group, University of Bremen, Bremen, Germany; 3 Institut für Diagnostische und Interventionelle Radiologie und Nuklearmedizin, Katholisches Klinikum Bochum, Universitätsklinikum der Ruhr Universität Bochum, Bochum, Germany

**Keywords:** Medical imaging, Image processing

## Abstract

Automatic liver tumor segmentation can facilitate the planning of liver interventions. For diagnosis of hepatocellular carcinoma, dynamic contrast-enhanced MRI (DCE-MRI) can yield a higher sensitivity than contrast-enhanced CT. However, most studies on automatic liver lesion segmentation have focused on CT. In this study, we present a deep learning-based approach for liver tumor segmentation in the late hepatocellular phase of DCE-MRI, using an anisotropic 3D U-Net architecture and a multi-model training strategy. The 3D architecture improves the segmentation performance compared to a previous study using a 2D U-Net (mean Dice 0.70 vs. 0.65). A further significant improvement is achieved by a multi-model training approach (0.74), which is close to the inter-rater agreement (0.78). A qualitative expert rating of the automatically generated contours confirms the benefit of the multi-model training strategy, with 66 % of contours rated as good or very good, compared to only 43 % when performing a single training. The lesion detection performance with a mean F1-score of 0.59 is inferior to human raters (0.76). Overall, this study shows that correctly detected liver lesions in late-phase DCE-MRI data can be automatically segmented with high accuracy, but the detection, in particular of smaller lesions, can still be improved.

## Introduction

Segmentation of the liver and liver tumors is a useful and sometimes necessary pre-processing step in the planning of many liver cancer therapies, such as radiofrequency ablation or selective internal radiation therapy (SIRT), but is also very time-consuming when performed manually. In recent years, deep learning (DL) methods have predominantly been used to solve these segmentation tasks with very high accuracy, in particular for the liver^1^. Automatic DL-based liver segmentation combined with manual corrections has shown to reduce the mean required interaction time compared to fully manual segmentation by 80 %, with a significantly lower inter-observer variability^2^. However, the segmentation of liver tumors remains challenging, due to the very high variability in shape, size, and overall appearance of the tumors.

Most recent segmentation research on fully automatic liver and liver tumor segmentation has focused on computed tomography (CT) image data, with a dedicated Liver Tumor Segmentation Benchmark (LiTS)^1^. As of 2017 when the LiTS challenge was held, the best performing methods, based on convolutional neural networks, reached a mean Dice score of 0.96 for liver segmentation and 0.70 for tumor segmentation. Since then, a wide range of algorithms and deep neural network architectures have been proposed or evaluated on the publicly available dataset, including self-configuring frameworks such as the nnU-Net^3^ or T-AutoML^4^. A full review of papers using the LiTS CT data is beyond the scope of this work.

From a clinical perspective, contrast-enhanced magnetic resonance imaging (MRI) shows significantly higher sensitivity compared to CT for the diagnosis of hepatocellular carcinoma (HCC) in patients with cirrhosis^5^. The higher accuracy of MRI for the detection and characterization of liver disease is mainly based on the higher soft tissue contrast as compared to CT, where contrast is generated only based on the different electron density of tissues. With MRI, different contrast mechanisms can be utilized to increase differentiation of various tissues, e.g., T1-, T2-, and diffusion-weighted sequences. Furthermore, using hepatocyte specific contrast agents, healthy liver cells can selectively be stained, while malignant tissue remains unstained and is displayed hypointense, i.e., dark. This increases the precision of volumetry of liver malignancies and with this allows higher accuracy in dose planning for SIRT. Therefore, MRI is often the imaging modality of choice for diagnosis and therapy planning. Clinical limitations of MRI are the longer acquisition time as compared to CT and that some implanted patient assistance devices, e.g., older pacemakers, are not MRI compatible.

From a technical perspective, automatic image processing of MRI data is usually considered more difficult than of CT data, because of the high heterogeneity in imaging protocols, dependence on the imaging device and non-standardized gray values. As a consequence, more effort for normalization of the image data is required. To the best of our knowledge, only few studies have applied deep learning techniques to the task of segmenting liver tumors or metastases in MRI data^6–10^, with segmentation performances in a similar Dice score range as in the LiTS challenge. In this study, we focus on segmentation of liver tumors in the late hepatocellular phase of dynamic contrast-enhanced MRI (DCE-MRI). Since no public benchmark test data are available for MRI tumor segmentation, we compare the automatic segmentation results to ground truth generated by three human raters, and adopt the use of uncertainty-aware evaluation scores^11^ that take the inter-rater agreement into account.

An additional technical challenge of deep learning methods in general is the non-repeatability of trainings, due to random effects that cannot all be controlled by using a fixed random seed^12^. Depending on the random weight initialization and further factors linked to the computing hardware, one and the same deep learning model can yield different results when trained multiple times on the same data. In this contribution, we propose to leverage this observation by following a multi-model training strategy, that starts by training sixteen models and narrows them down to the best one, using the Hyperband method^13^. The final model, which is chosen based on validation data, significantly outperforms a single trained model on an independent test set, and achieves a higher average qualitative rating.

## Methods

### Image data

We used DCE-MRI data of 107 patients with primary liver cancer or liver metastases acquired on a 3T Discovery MRI scanner (GE Healthcare Systems, USA). The late hepatocellular phase acquired 15 minutes after the injection of contrast agent Gd-EOB-DTPA (Primovist, Bayer Healthcare, Germany) was used, as it provides good contrast between liver parenchyma and tumor or surrounding tissue. The original in-plane image resolution ranged from 0.74 to 1.76 mm and the slice thickness from 2 to 5 mm. The data was split into 58 cases (71 %) for training, 5 cases (6 %) for validation, and 19 cases (23 %) for testing, with percentages based on 82 contoured cases in total. An additional 25 cases were available for a qualitative evaluation without reference contours. Imaging data for this study was evaluated after approval by the ethics committee of Sächsische Landesärztekammer (EK-BR-79/16-1 / 118834). All patients gave written informed consent. We hereby confirm that all experiments were performed in accordance with the relevant guidelines and regulations.

### Image pre-processing and augmentation

The in-plane resolution was resampled to 1 mm, but there was no resampling between slices to avoid resampling artifacts of smaller lesions. This means that the final voxel size was $1\times 1\times z\text { mm}^3$ where *z* is the original slice spacing of each image. Image gray values were normalized by linearly mapping the 2^nd^ and 98^th^ gray value percentiles, computed within a liver mask, to 0 and 1 without clipping values outside this interval. All pre-processing was performed in MeVisLab^14^ (MeVis Medical Solutions, Germany). The extracted patches (see Sect. *Deep neural network*) were augmented on-the-fly using the following three transforms: flipping along all image axes with a 50 % probability per axis, linear rescaling of the voxel value distance from the gray value mean using a random factor $\alpha$ with values uniformly sampled from the interval $\left[ 0.75,1.25\right)$ (thereby adjusting the contrast), and shifting of the gray value mean by a normally distributed value $\beta$ with mean 0 and standard deviation 0.25.

### Reference contours

The liver and liver lesions were contoured for 82 cases by a medical radiology assistant (denoted rater R_1_) with over ten years of experience using a semi-automatic contouring tool^15^. The 19 test cases were additionally segmented by a radiologist in training with 2 years of experience (denoted R_2_) and a senior radiologist with 21 years of experience (denoted R_3_), using a commercial contouring tool (Varian Eclipse, Contouring, Varian Medical Systems, USA). In all cases, reference contours were drawn using the late hepatocellular phase. For 25 cases, no contours were provided, these cases were used in a qualitative rating of the automatic segmentations in addition to the annotated test data.

The liver contours were only used for pre-processing, post-processing and patch sampling. The training itself was a binary task of distinguishing background (including liver parenchyma) from lesion foreground voxels.

### Deep neural network

**Figure 1:**
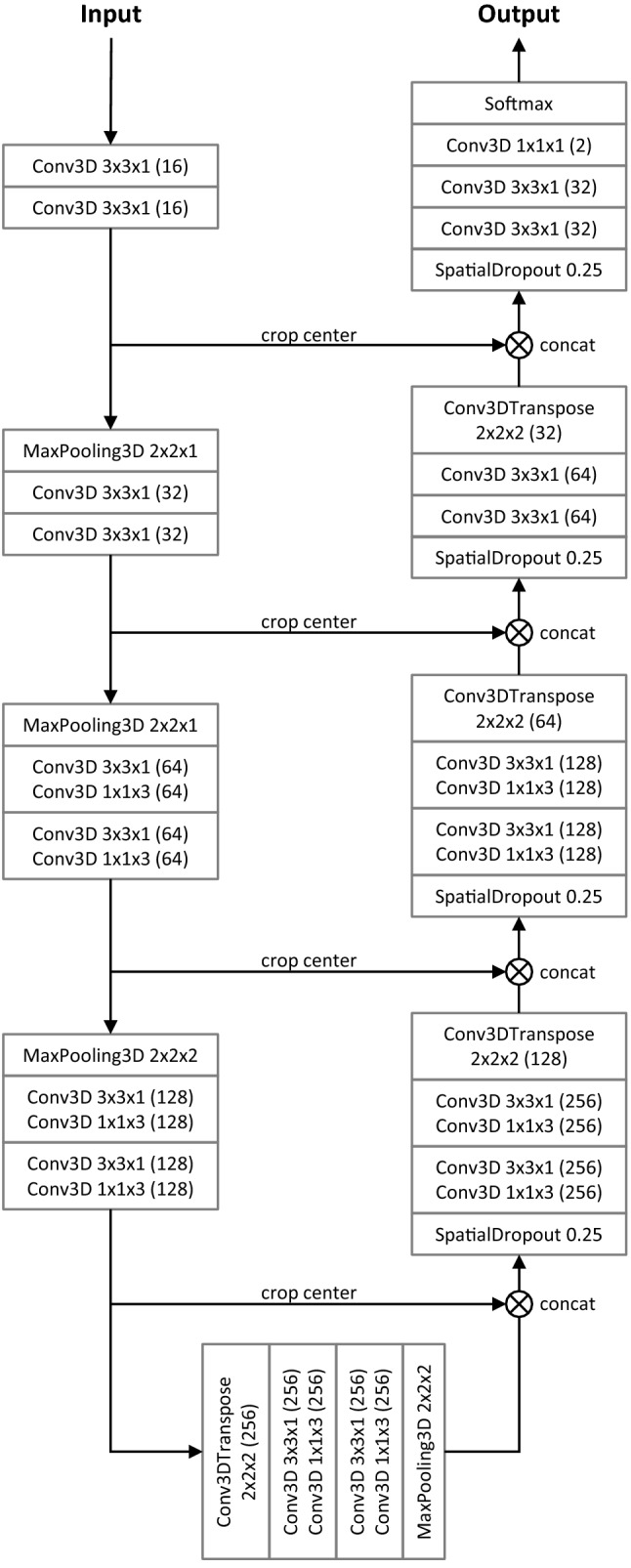
Diagram of the anisotropic U-Net (aU-Net) introduced by Chlebus et al.^16^. All convolutional blocks except for the last one are followed by batch normalization and ReLU activation function. Numbers in brackets indicate the number of features generated by each layer. All convolutions are unpadded, so that cropping of the feature map centers is required before concatenation. Downsampling is performed by max pooling, upsampling by transposed convolutions.

The deep neural network architecture, depicted in Fig. 1, was an anisotropic version of the 3D U-Net, hence it is referred to as “aU-Net”^16,19^. The anisotropy refers to the different number of convolutions in *xy* (in-plane) vs. *z* (between slices), which is suitable given the anisotropy of the processed image data. The baseline architecture was a 2D U-Net^17^ with five resolution levels and $3\times 3\times 1$ convolutions, followed by a $1\times 1\times 3$ convolution in the three lowest resolution levels only. The main motivation of this approach is to mimic the way that radiologists inspect volumetric data, by not only including in-plane information but also few adjacent slices^19^. Because of the reduced number of 3D convolutions, the aU-Net has less parameters than a full 3D U-Net and also requires smaller input patches, making it easier to train and allowing for more resolution levels and therefore a larger receptive field in-plane. The concept of training with an anisotropic receptive field has also previously been described for the purpose of multi-organ segmentation^18^. We used batch normalization^20^ and dropout^21^ for regularization.

In our application, we assume that the liver mask is generated by a separate CNN and manually corrected in a preceding step. Therefore, we use the available liver contours for focusing the training on the liver region by applying a liver weight mask to the loss and by only sampling those patches that include the liver. In detail, the aU-Net was trained on the binary task of distinguishing liver lesions from background, which included the liver parenchyma. We used the Dice loss^22^, computed within a dilated liver mask only, the Adam optimizer^23^ and batch size 2. In the network architecture, we used convolutions without zero-padding, meaning that the image or feature map size is reduced with each convolution. To account for this reduction, we input larger patches ($236\times 236\times 72$) into the network than we obtained at the output ($52\times 52\times 32$), i.e. using a symmetric padding of $92\times 92\times 20$ voxels.

The patches were sampled so that 90 % included at least one tumor voxel and the remaining 10 % included at least one liver voxel, to restrict the sampled patches to the relevant liver region. For training, all non-tumor liver voxels were assigned to the background class. We used the Jaccard coefficient, computed every 500 iterations on the validation data, as validation metric for choosing the best network parameter state for the final model. All training was performed using the in-house deep learning infrastructure *RedLeaf*^24^ with a Keras^25^ backend.

### Multi-model training

The neural network training involves several random components, such as the random weight initialization following a specific probability distribution in the convolutional kernels or the order of the sampled patches. In order to reduce the chance of training a subpar model, we employed a method called Hyperband^13^, implemented in the BHOB package^26^. Using this approach, we started by training $2^4$ models, all of them used the default weight initialization distributions defined in Keras (Glorot uniform for convolutional layers), but different random seeds, leading to differently initialized CNNs. After 5k, 10k, 20k, and 40k iterations, the number of models was reduced by half, based on the highest validation Jaccard values. The remaining model was trained until iteration 80k. We did not optimize any hyper-parameters of the model.

To estimate the benefit of the multi-model training, we also trained a single aU-Net using only one random seed for the weight initialization. We refer to the two resulting algorithms as $A_1$ (with multi-model training) and $A_2$ (without multi-model training).

### Post-processing

The raw model output was restricted to the liver mask and binarized by thresholding at 0.5. In validation experiments, we sometimes observed thin false positives at the liver border, typically in places where a large hypo-intense structure such as a large vessel was directly adjacent to the liver. To remove such false segmentations, a simple post-processing using morphological operations was applied: The liver mask was eroded using a $7\times 7$ kernel to generate a mask of the liver border by subtracting the eroded from the original mask. Within the liver border region, the binary tumor mask was filtered with an opening operation using a $3\times 3$ kernel to remove thin structures.

### Evaluation measures

We quantitatively evaluated the trained algorithms with respect to two criteria: segmentation and detection performance, taking the three human raters into account. Since a quantitative evaluation may not represent a clinical usabilty or usefulnees, we performed an additional clinical expert rating.

#### Segmentation performance

We computed the Dice score *D* for algorithms $A_1$ and $A_2$ against each human rater $R_i$, denoted $D(A_j,R_i)$, as well as the mean across raters $\overline{D(A_j,R_i)}$ per algorithm $A_j$. For comparison, we computed the mean of all pair-wise Dice scores between human raters $\overline{D(R_i,R_j)}$. Based on these measures, we computed an uncertainty-aware score

1 $$\begin{aligned} \phi (A,R_1,R_2,R_3)&= \max \left( 1-\alpha \frac{{\hat{\epsilon }}(A,R_1,R_2,R_3)}{{\bar{\epsilon }}(R_1,R_2,R_3)},0\right) \end{aligned}$$

with

2 $$\begin{aligned} {\hat{\epsilon }}(A,R_1,R_2,R_3)&= 1-\overline{D(A,R_i)} \quad \text {and}\quad {\bar{\epsilon }}(R_1,R_2,R_3)=1-\overline{D(R_i,R_j)} \end{aligned}$$

as proposed by Moltz^11^ for a discrepancy measure $\epsilon$. It is based on a score to evaluate liver and liver tumor segmentation in a *MICCAI 2007 Grand Challenge* for liver segmentation^27^, but furthermore puts the mean algorithm performance in relation to the inter-observer-variability. Using $\alpha =0.1$, a value of 0.9 or above indicates that the automatic segmentation is considered as good as the manual segmentation, taking the uncertainty among observers into account.

#### Detection performance

There may be no one-to-one correspondence between lesions in reference mask and predicted mask, due to splitting or merging of lesion instances. Therefore, we first established *N* : *M* correspondences between lesions in the two masks using an algorithm and implementation by Chlebus et al.^28^: Each correspondence is made up of *N* lesions in the reference mask and *M* lesions in the predicted mask. The lesions are clustered into correspondences, so that the per-correspondence Dice score is maximized. If the per-correspondence Dice score is larger than 0.2, the correspondence is counted as true positive, otherwise as false positive and false negative. All lesions in the reference or predicted masks, that are not part of any correspondence, are counted as false negative or false positive respectively. Based on these counts, we computed recall, precision, and F1-score per test case, as well as the number of false positives per case (FPC).

#### Expert rating

A qualitative rating of the automatic tumor segmentation quality was performed by a senior radiologist (R3) on 44 (19 annotated + 25 additional) test cases. Each contour was rated on a scale from 1 (very poor segmentation) to 5 (very good segmentation). During the rating, the expert was blinded to the algorithm: In a first round, all cases were shown to the rater with the automatic segmentation from either algorithm $A_1$ or $A_2$ (assigned randomly per case). In a second round, the cases were presented in the same order, and the second segmentation, not shown in the first round, was presented. In both rounds, the rater was asked to rate the image quality on a scale from 1 (poor quality) to 3 (good quality), where the mean of both ratings was used for the evaluation.

#### Statistical tests

We used the Wilcoxon-signed rank test^29^ ($\alpha =0.05$) implemented in SciPy^30^ for statistical significance testing of differences in segmentation and detection performance of algorithms and raters. The Benjamini-Hochberg method^31^ with false discovery rate 0.05 was applied to account for multiple testing during segmentation and detection evaluation respectively.

## Results

### Segmentation performance

**Figure 2:**
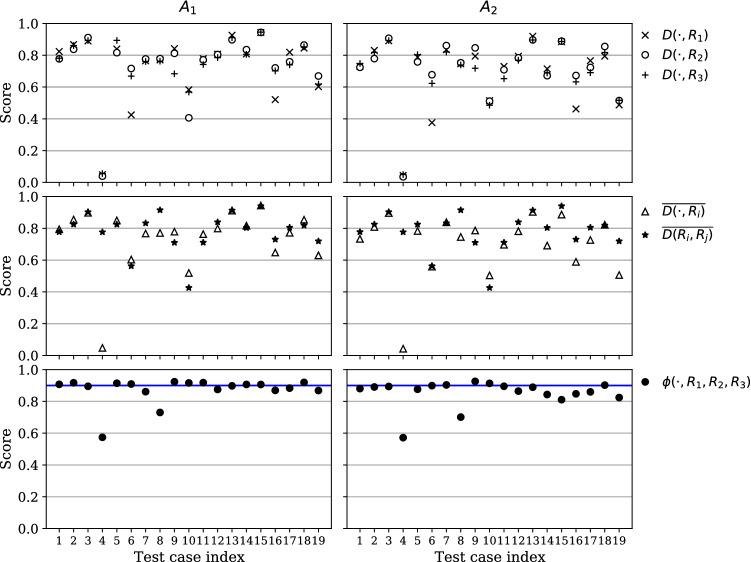
Quantitative evaluation of liver tumor segmentation. Shown are the Dice score per test case for each rater (top row), average Dice score across raters and inter-rater Dice score (middle row), and uncertainty-aware score (bottom row), where a value of 0.9 corresponds to human-level performance.

Figure 2 shows the segmentation scores per algorithm and test case, with a summary in Table 1. Algorithm $A_1$, that was based on multi-model training, significantly outperforms algorithm $A_2$ with respect to all raters and metrics. For most test cases, the mean agreement of $A_1$ with all raters, $\overline{D(A_1,R_i)}$, is close to the inter-observer-variability measured by $\overline{D(R_i,R_j)}$. This results in high uncertainty-aware scores $\phi (A_1,R_1,R_2,R_3)$ with mean and standard deviation $0.873 \pm 0.08$, just below threshold of 0.9 that is considered similar to human-level performance, by design of the metric. For both algorithms, there are only two test cases for which the uncertainty-aware score deviates considerably from 0.9. For the inter-observer-variability, we find a mean agreement of $\overline{D(R_i,R_j)} = 0.781 \pm 0.121$ in the Dice score, which is still slightly higher than the average performance of $A_1$ at $0.738\pm 0.194$.

**Figure 3:**
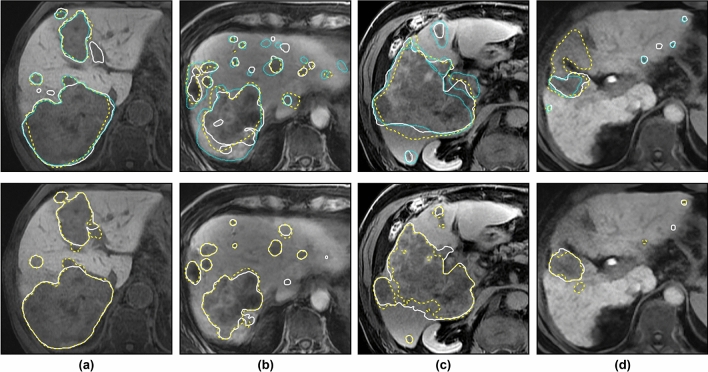
Exemplary test cases with manual and automatic liver tumor segmentations. Reference segmentations (top row) and automatic segmentation results (bottom row) are shown for test cases 13, 17, 14 and 10 (left to right) in Fig. 2. In the top row, white solid / yellow dashed / cyan finely dotted contours correspond to raters $R_1$ / $R_2$ / $R_3$. In the bottom row, white solid / yellow dashed contours correspond to algorithms $A_1$ / $A_2$. Based on the average performance of $A_1$ against all raters $\overline{D(A_1,R_i)}$, these cases represent the (a) upper, (b, c) median, and (d) lower end of the Dice score range, not including outlier case 4.

Figure 3 shows reference and automatic segmentations for representative example slices of four different test cases on the upper, median and lower end of the Dice score range for the comparison of $A_1$ against all raters, not including outlier case 4 in Figure 2. For the correctly detected lesions in cases (**a**–**c**), the automatic segmentations are qualitatively similar to the reference segmentations. In case (**d**), 11 small false positives and 22 small false negatives across the slices (not all visible in the figure) contribute to a decrease of the Dice score, even though the largest lesion is well delineated with respect to $R_1$ and $R_3$. False positive and negative detections remain a main source of error, as discussed in the next section.

**Table 1:** Summary of quantitative evaluation of liver tumor segmentation. Mean and standard deviation of the different scores for segmentation evaluation are given for algorithms $A_1$ and $A_2$. For comparison, the mean Dice score among raters is $\overline{D(R_i,R_j)} = 0.781 \pm 0.121$. The *p*-value indicates statistical significance of differences between both algorithms, also after correction for multiple testing.

	$D(\cdot ,R_1)$	$D(\cdot ,R_2)$	$D(\cdot ,R_3)$	$\overline{D(\cdot ,R_i)}$	$\phi (\cdot ,R_1,R_2,R_3)$
$A_1$	0.732 ± 0.210	0.744 ± 0.200	0.738 ± 0.189	0.738 ± 0.194	0.873 ± 0.082
$A_2$	0.689 ± 0.214	0.714 ± 0.195	0.697 ± 0.190	0.700 ± 0.196	0.852 ± 0.082
*p*-value	0.0017	0.0269	0.0029	0.0025	0.0022

### Detection performance

Table 2 summarizes the detection performance of both algorithms compared to all raters, as well as the inter-observer-variability. Algorithm $A_1$ leads to lower recall but higher precision than $A_2$, and overall seems to achieve slightly higher F1-scores. However, none of these differences are significant after correction for multiple testing. $A_1$ has a median FPC of 2.3 averaged across all raters, compared to 6 FPC for $A_2$, and 1 FPC for the inter-rater comparison. Overall, the automatic methods do not reach the performance of the inter-rater comparison. Figure 3 illustrates, that in particular smaller false positives and false negatives contribute to the decrease in detection scores.

**Table 2:** Summary of quantitative evaluation of liver tumor detection. Mean and standard deviation of recall, precision and F1-Score, and median FPC are given for algorithms $A_1$ and $A_2$ evaluated against each rater, as well as of the mean per test case ($\overline{R_i}$). The last row shows the mean pair-wise comparison of raters $\overline{(R_i,R_j)}$.

	Recall		Precision		F1-Score		FPC	
	$A_1$	$A_2$	$A_1$	$A_2$	$A_1$	$A_2$	$A_1$	$A_2$
$R_1$	0.699 ± 0.305	0.726 ± 0.294	0.533 ± 0.256	0.387 ± 0.185	0.549 ± 0.210	0.463 ± 0.181	3	6
$R_2$	0.680 ± 0.300	0.736 ± 0.308	0.590 ± 0.270	0.436 ± 0.248	0.595 ± 0.251	0.497 ± 0.233	3	6
$R_3$	0.751 ± 0.277	0.781 ± 0.288	0.574 ± 0.249	0.430 ± 0.237	0.623 ± 0.228	0.510 ± 0.216	2	6
$\overline{R_i}$	0.710 ± 0.268	0.748 ± 0.269	0.566 ± 0.225	0.418 ± 0.205	0.589 ± 0.201	0.490 ± 0.192	2.3	6
$\overline{(R_i,R_j)}$	0.804 ± 0.175		0.787 ± 0.1805		0.761 ± 0.165		1	

### Expert rating

**Figure 4:**
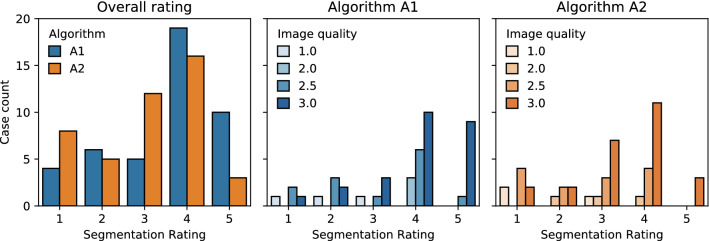
Histogram of the qualitative segmentation rating. The rating was performed on 44 test cases using a scale from 1 (very poor segmentation) to 5 (very good segmentation). The center and right plot show the rating depending on the mean image quality rating on a scale from 1 (poor quality) to 3 (good quality) from two rounds of rating.

Histograms of the qualitative segmentation rating are shown in Fig. 4. Algorithm *A*_1_ achieves an average rating of 3.6 (median 4) with a good or very good quality in 66 % of cases. In comparison, algorithm *A*_2_ only achieves an average rating of 3.0 (median 3) with 43 % of good or very good ratings. Low image quality leads to overall lower ratings. These qualitative results confirm the superior performance of *A*_1_ from the quantitative evaluation.

### Comparison to results from literature

As outlined in the introduction, MRI-based liver tumor segmentation is less studied than CT-based segmentation and no public challenge datasets are available for evaluation. This makes a comparison of methods more difficult than for CT data, for which the LiTS 2017 challenge^1^ reports a maximum Dice score performance of 0.67 (ISBI) and 0.70 (MICCAI) for tumor segmentation. These results are slightly below but in a similar range as the performance on MRI reported in this study.

The study with highest comparability to our results was performed by Chlebus et al.^7^, who reported a mean Dice score of $0.647\pm 0.210$. A subset of the same internal data set was used as in this study with segmentations of rater $R_1$, though using a different training/validation/test split and a 2D U-Net architecture. As in this study, only the late hepatocellular phase of a LAVA DCE-MRI sequence of 57 cases was used for training. The improved mean performance of $0.732\pm 0.210$ (based on $R_1$) presented here, indicates a benefit of using the aU-Net and multi-model training.

Christ et al.^6^ achieved a mean Dice score of 0.697, using 31 cases in total. They applied a cascade of two U-Nets for first segmenting the liver and subsequently the liver lesions only within the liver ROI. They used a 2D architecture followed by a conditional random field, and completely different MRI sequences as input (diffusion-weighted MRI) than in this study.

Jansen et al.^8^ used all phases of DCE-MRI of 121 patients to segment liver metastases and achieved an average recall of 0.645 and a median of 5 false positives per case, which is roughly similar to our detection results using $A_1$. They did not evaluate the segmentation performance using Dice scores or other metrics. By adding diffusion-weighted data and a second pathway to their fully convolutional network architecture, they were able to detect almost all lesions with a median of only 2 false positives per case. This demonstrates the benefit of using additional image information for liver lesion detection, to increase both precision and recall. In our study, diffusion weighted image data was not available for the majority of patients.

Bousabarah et al.^9^ used three contrast phases of DCE-MRI of 174 patients to simultaneously segment liver and HCC using a 2D U-Net, reaching a mean Dice score of 0.68. In their study, post-processing using a random forest classifier and cluster thresholding reduced the average false positive rate from 2.81 to 0.62 FPC, while maintaining a recall of 73 %.

Zhao et al.^10^ used non-contrast MRI (T1FS, T2FS, DWI) of 255 subjects with HCC or hemangioma to detect and segment liver lesions. They also made use of contrast-enhanced MRI, however only during training within a proposed Radiomics-guided adversarial loss. Their method achieved a mean Dice score of 83.6 compared to 78.9 for a U-Net baseline, and a detection accuracy of 93 %. They also demonstrated a small decrease in performance when omitting one or more MRI sequences or contrast phases, suggesting that using complementary imaging information can improve lesion detection and segmentation.

### Runtime of the multi-model training approach

**Figure 5:**
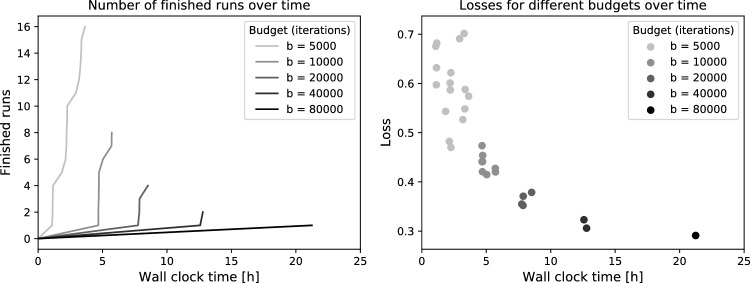
Runtime and losses of the multi-model training. Shown are the number of finished runs (left) and the corresponding losses (right) achieved within a total wall clock time of 21:12 hours using parallel workers. The plots are based on the hpbandster.visualization python package^26^.

For practical use, a multi-model training approach is only feasible if the runtime of training and inference is reasonable compared to a single training. As the best model is chosen as final result and no model ensembling is performed, the inference time does not increase with the multi-model training approach. In our set-up, multi-model training increased the total number of iterations by a factor of 3 from 80k to 240k iterations. On our GPU cluster, this equaled 2 days and 4:23 hours GPU time compared to 16:57 hours for a single model. Using parallel workers, the wall clock time were 21:12 hours, as shown in Fig. 5. The figure also shows the variation in losses after the first training stage, which range from 0.47 to 0.70.

## Discussion

Our results indicate that the proposed approach of multi-model training improves the liver lesion segmentation performance quantitatively as well as qualitatively. With an increase in total training time by a factor of 3, that can however be parallelized to a large degree, and no increase in inference time, multi-model training appears to be a simple approach to improve the segmentation performance. In this study, the model selection was performed on a small validation set consisting of only five cases. A larger and more representative validation set might further improve the results and make the method more robust, due to a reduced risk of overfitting to the validation data. As no hyper-parameters have been tuned, the final model of the multi-model training seems to simply have won the “initialization lottery”, as formulated by Frankle and Carbin in the context of model pruning^32^.

Compared to liver segmentation, which often reaches Dice scores above 0.95 for both CT^1^ and MRI^7^, the mean Dice scores for liver tumor segmentation are lower, typically below 0.8. Our use of uncertainty-aware scores puts this into perspective: in many cases where the Dice score of the automatic method is low, the deviation between raters is high. For example, note that the poorly performing case d) in Fig. 3 still gets an uncertainty-aware score above 0.9, as can be seen in Fig. 2 (case index 10), because of the high inter-rater variance for this case. In terms of segmentation performance, our best algorithm (mean Dice score 0.74) is close to the inter-rater agreement (mean Dice score 0.78). The remaining challenge in liver lesion segmentation seems to be the correct detection of smaller lesions, in terms of both precision and recall. In this study, the main source of false positives were small vessels or partial volume effects of other structures that were segmented as lesions. The use of post-processing methods^9,28^ or of more contrast phases and sequences^8,10^ could further improve our results with respect to the detection performance. However, in clinical routine imaging, not all sequences, such as DWI, may be available. For future work, a two-stage approach separating detection and segmentation into two distinct tasks might further improve the overall performance, and recall in particular. Alternatively, dedicated architectures for instance detection and segmentation, such as Mask R-CNN^33^, could prove to be beneficial. An accurate detection is very relevant from a clinical perspective, as the distribution of lesions within the liver is important for the choice of possible therapies and interventions.

We did not perform an ablation study for estimating the benefit of using an anisotropic 3D U-Net architecture compared to a standard 3D or 2D U-Net. However, compared to a previous study on a partially identical dataset using 2D U-Nets^7^, the chosen anisotropic 3D architecture improves the mean Dice score from 0.65 to 0.70 (without multi-model training) and 0.74 (with multi-model training). This suggests that both the architecture as well as the multi-model training approach contribute to an improved segmentation performance compared to previously published results.

In general, the direct quantitative comparison with other segmentation methods is difficult, as no public training and test dataset is available for MRI-based liver lesion segmentation. Furthermore, different studies focus on different clinical problems such as segmentation of HCC tumors versus metastases or both, as in our study. The difficulty of these tasks is also influenced by the heterogeneity of the dataset and by the available MRI sequences and contrast phases. In this study, we used a study dataset from a single site with a fixed imaging sequence, which results in a comparably homogeneous dataset and therefore in an easier task. In future work, the robustness of the proposed methods should be evaluated on multi-centric data.

## Conclusion

We demonstrated the effectiveness of an anisotropic U-Net and multi-model training for the task of liver tumor segmentation in MRI data. The resulting model yields a segmentation performance that is close to the inter-rater-agreement of three clinical experts, but could further be improved with respect to detection of smaller lesions in particular.

## Data Availability

Evaluation results can be found within the manuscript and on Figshare at the following link: https://doi.org/10.6084/m9.figshare.19189316. Image data used in this paper cannot be shared publicly due to legal reasons (it would compromise patient confidentiality). Queries for image data access can be filed to the ethics board of the Sächsische Landesärztekammer (https://www.slaek.de).
